# The epidemiological study of rescue missions following drowning accidents at the pre-hospital emergency system of Mazandaran province from March 23 to September 22, 2017

**Published:** 2019-07

**Authors:** Zoya Hadinejad, Kobra Qolami, Saeedeh Yousefi, Hasan Talebi, Seyede-Masoomeh Hashemi

**Affiliations:** ^*a*^Disaster and Emergency Medical Management Center, Mazandaran University of Medical Sciences, Iran.

## Abstract

**Background:**

Drowning is one of the major health problems that is often neglected. Given that Mazandaran province is located on the banks of the Caspian Sea, and many people travel the northern provinces of Iran, and insufficient information is found on the number of rescue missions following the drowning accidents in the pre-hospital emergency domain, this study aimed to investigate the epidemiological status of the drowned cases, and design better plans for the prevention and control of these missions.

**Methods:**

In this retrospective cross-sectional study, the mission forms related to all drowned cases in the pre-hospital emergency departments of Mazandaran from March 23 to September 22, 2017 were reviewed and the data including age, sex, drowning place, outcome of the mission (dispatched and cancelled missions, outpatient treatment, death, among others), the date of the accident, and being native or non-native drowned person were extracted. The data were analyzed using SPSS (version 19) and Fischer and Chi-square tests.

**Results:**

Out of 219 drownings from March 23 to September 22, 2017 recorded by the Emergency Department of Mazandaran University of Medical Sciences, 159 cases (72.6%) were male and 60 cases (27.4) female. The mean age of the drowned individuals was 29±3 years old, and most accidents happened for 25-29 year-old ones. Drowning occurred mostly in places outside the patrolled area with 190 cases (86.7%), as well as in summer with 185 cases (84.5%) (from 23 July to 22 August) with 77 cases. The patients were sent to health centers in 43.8% of the missions. The mean age of 39 dead ones was 39±2 years old.

**Conclusions:**

Most of the drowning cases occurred outside of the patrolled area in the young and productive age group of society. Therefore, it seems necessary to establish and develop sea-protected shores, as well as provide proper education.

**Keywords:**

Drowning, Pre-hospital emergency, Epidemiology

